# Adversarial counterfactual augmentation: application in Alzheimer’s disease classification

**DOI:** 10.3389/fradi.2022.1039160

**Published:** 2022-11-30

**Authors:** Tian Xia, Pedro Sanchez, Chen Qin, Sotirios A. Tsaftaris

**Affiliations:** ^1^ School of Engineering, University of Edinburgh, Edinburgh, United Kingdom; ^2^ The Alan Turing Institute, London, United Kingdom; ^3^ Department of Electrical and Electronic Engineering, Imperial College London, London, United Kingdom

**Keywords:** Alzheimer’s disease, generative model, classification, counterfactuals, data efficiency

## Abstract

Due to the limited availability of medical data, deep learning approaches for medical image analysis tend to generalise poorly to unseen data. Augmenting data during training with random transformations has been shown to help and became a ubiquitous technique for training neural networks. Here, we propose a novel adversarial counterfactual augmentation scheme that aims at finding the most *effective* synthesised images to improve downstream tasks, given a pre-trained generative model. Specifically, we construct an adversarial game where we update the input *conditional factor* of the generator and the downstream *classifier* with gradient backpropagation alternatively and iteratively. This can be viewed as finding the ‘*weakness*’ of the classifier and purposely forcing it to *overcome* its weakness via the generative model. To demonstrate the effectiveness of the proposed approach, we validate the method with the classification of Alzheimer’s Disease (AD) as a downstream task. The pre-trained generative model synthesises brain images using age as conditional factor. Extensive experiments and ablation studies have been performed to show that the proposed approach improves classification performance and has potential to alleviate spurious correlations and catastrophic forgetting. Code: https://github.com/xiat0616/adversarial_counterfactual_augmentation

## 1. Introduction

Deep learning has been playing an increasingly important role in medical image analysis in the past decade, with great success in segmentation, diagnosis, detection, etc (1). Although deep-learning based models can significantly outperform traditional machine learning methods, they heavily rely on the large size and quality of training data (2). In medical image analysis, the availability of large dataset is always an issue, due to high expense of acquiring and labelling medical imaging data (3). When only limited training data are available, deep neural networks tend to memorise the data and cannot generalise well to unseen data (4, 5). This is known as *over-fitting* (4). To mitigate this issue, data augmentation has become a popular approach. The aim of data augmentation is to generate additional data that can help increase the variation of the training data.

Conventional data augmentation approaches mainly apply random image transformations, such as cropping, flipping, and rotation etc. to the data. Even though such conventional data augmentation techniques are general, they may not transfer well from one task to another (6). For instance, color augmentation could prove useful for natural images but may not be suitable for MRI images which are presented in greyscale images (3). Furthermore, traditional data augmentation methods may introduce *distribution shift*, i.e., the change of the joint distribution of inputs and outputs, and consequently adversely impact the performance on non-augmented data during inference^1^ (i.e., during the application phase of the learned model) (7).

Some recently developed approaches learn parameters for data augmentation that can better improve downstream task, e.g. segmentation, detection, diagnosis, etc., performance (6, 8, 9) or select the hardest augmentation for the target model from a small batch of random augmentations for each traning sample (10). However, these approaches still use conventional image transformations and do not consider semantic augmentation (11), i.e., creating unseen samples by changing semantic information of images such as changing the background of an object or changing the age of a brain image. Semantic augmentation can complement traditional techniques and improve the diversity of augmented samples (11).

One way to achieve semantic augmentation is to train a deep generative model to create *counterfactuals*, i.e., synthetic modifications of a sample such that some aspects of the original data remain unchanged (12–16). However, these approaches mostly focus on the training stage of generative models and randomly generate samples for data augmentation, without considering which counterfactuals are *more effective* for downstream tasks, i.e. data-efficiency of the generated samples. Ye et al. (17) use a policy based reinforcement learning (RL) strategy to select synthetic data for augmentation with reward as the validation accuracy. Xue et al. (18) propose a cGAN based model to augment classification of histopathology images with a selective strategy based on assigned label confidence and feature similarity to real data. By contrast, our approach focuses on finding the weakness (i.e. the hard counterfactuals) of a downstream task model (e.g. a classifier) and forces it to overcome its weakness. Similarly, Ye et al. (17) use a policy based reinforcement learning (RL) strategy to select synthetic data for augmentation, with reward as the validation accuracy, but the instability of RL training could perhaps hinder the utility of their approach. Wang et al. (11), Li et al. (19), Chen and Su (20) proposed to augment the data in the latent space of the target deep neural network, by estimating the covariance matrix of latent features obtained from latent layers of the target deep neural network for each *class* (e.g., car, horse, tree, etc.) and sampling directions from the feature distributions. These directions should be semantic meaningful such that changing along one direction can manipulate one property of the image, e.g. color of a car. However, there is no guarantee that the found directions will be semantically meaningful, and it is hard to know which direction controls a particular property of interest.

**Figure 1:**
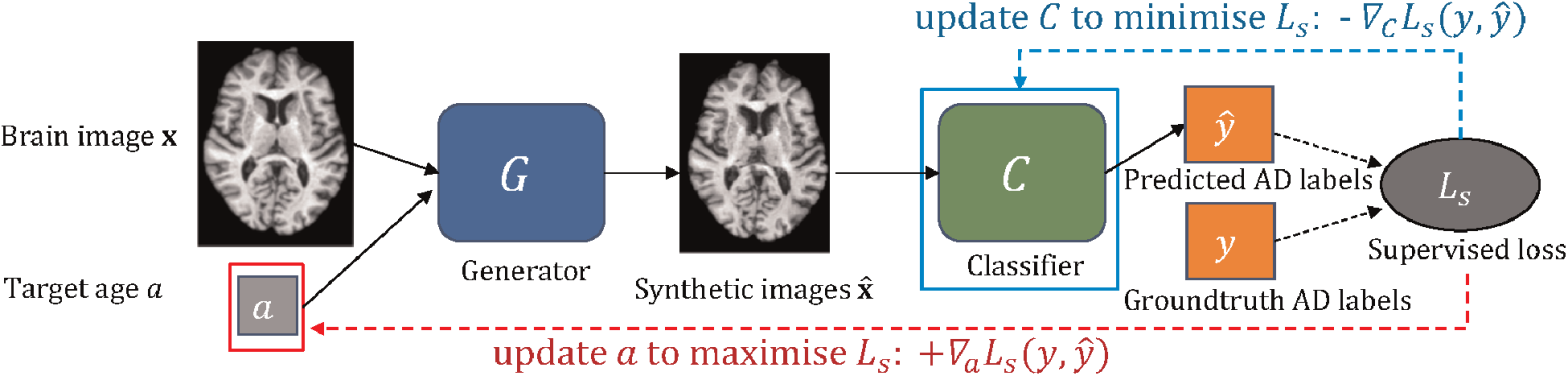
A schematic of the adversarial classification training. The pre-trained generator G takes a brain image x and a target age a as input and outputs a synthetically aged image $\hat{x}$ that corresponds to the target age a. The classifier C aims to predict AD label for a given brain image. To utilise G to improve C, we formulate an adversarial game between a (in red box) and C (in cyan box), where a and C are updated alternatively and iteratively using $L_{1}$ and $L_{2}$, respectively (see Section 2.3). Note G is frozen.

In this work, we consider the scenario that we have a classifier which we want to improve (e.g. an image-based classifier of Alzheimer’s Disease (AD) given brain images). We are also given some data and a pre-trained generative model that is able to create new data given an image as input and conditioning factors that can alter corresponding attributes in the input. For example, the generative model can alter the brain age of the input. We propose an approach to guide a pre-trained generative model to generate the *most effective* counterfactuals via an adversarial game between the input *conditioning factor* of the generator and the downstream classifier, where we use gradient backpropagation to update the *conditioning factor* and the *classifier* alternatively and iteratively. A schematic of the proposed approach is shown in Figure 1.

Specifically, we choose the classification of AD as the downstream task and utilise a pre-trained brain ageing synthesis model to improve the AD classifier. The brain ageing generative model used in this paper is adopted from a recent work (21), which takes a brain image and a target age as inputs and outputs an aged brain image.^2^ We show that the proposed approach can improve the test accuracy of the AD classifier. We also demonstrate that it can be used in a *continual learning*^3^ context to alleviate *catastrophic forgetting*, i.e. deep models forget what they have learnt from previous data when training on new given data, and can be used to alleviate *spurious correlations*, i.e. two variables appear to be causally related to one another but in fact they are not. Our contributions can be summarised as follows:

1. We propose an approach to utilise a pre-trained generative model for a classifier via an adversarial game between *conditional input* and the *classifier*. To the best of our knowledge, this is the first approach that formulates such an adversarial scheme to utilise pre-trained generators in medical imaging.
2. We improve a recent brain ageing synthesis model by involving Fourier encoding to enable gradient backpropagation to update *conditional factor* and demonstrate the effectiveness of our approach on the task of AD classification.
3. We consider the scenario of using generative models in a *continual learning* context and show that our approach can help alleviate *catastrophic forgetting*.
4. We apply the brain ageing synthesis model for brain *rejuvenation* synthesis and demonstrate that the proposed approach has the potential to alleviate *spurious correlations*.

## 2. Methodology

### 2.1. Notations and problem overview

We denote an image as x∼X, and a conditional generative model G that takes an image x and a conditional vector v as input and generates a counterfactual $\hat{x}$ that corresponds to v: $\hat{x}=G(x,v)$. For each x, there is a label y∼Y. We define a classifier C that predicts the label $\hat{y}$ for given x: $\hat{y}=C(x)$. In this paper, x is a brain image, y is the AD diagnosis of x, and v represents the target age a and AD diagnosis on which the generator G is conditioned. We select age and AD status to be conditioning factors as they are major contributors to brain ageing. We use a 2D slice brain ageing generative model as G, and a VGG^4^-based (22) AD classification model as C. In Xia et al. (21), the brain ageing generative model is evaluated in multiple ways, including several quantitative metrics: Structural Similarity (SSIM), Peak Signal-to-Noise Ratio (PSNR) and Mean Squared Error (MSE) between the synthetically aged brain images and the ground-truth follow-up images, and Predicted Age Difference (PAD), i.e. difference between the predicted age by a pre-trained age predictor and the desired target age. For more details of the evaluation metrics, please refer to Xia et al. (21), Section 4. Note that we only change the target age a in this paper, thus we write the generative process as $\hat{x}=G(x,a)$ for simplicity.

Suppose a pre-trained G and a C are given, the question we want to answer is: “*How can we use G to improve C in a (data) efficient manner*”? To this end, we propose an approach to utilise G to improve C via an adversarial game with *gradient backpropagation* to update a and C alternatively and iteratively.

### 2.2. Fourier encoding for conditional factors

The proposed approach requires backpropagation of gradient to the conditional factor to find the hard counterfactuals. However, the original brain ageing synthesis model (21) used *ordinal encoding* to encode the conditional age and AD diagnosis, where the encoded vectors are discrete in nature and need to maintain a certain shape, which hinders gradient backpropagation to update these vectors. Imagine a 5-dimensional ordinal vector representing the number 3 as [1,1,1,0,0]. If we compute gradients with respect to the vector to update it, and the gradients multiplied by alpha happen to be [ −0.3, −0.1, 0.1, 0.2, − 0.3] (for example), then the resulting vector would be [0.7,0.8,1.1,0.2,  −0.3], which is not a ordinal vector anymore. Converting this to obey ordinal rules will require that we first quantize to 0/1 and then check for ordinal order preservation of the 1 digits. Both are not easily differentiable.

To enable gradient backpropagation to update the conditional vectors, we propose to use *Fourier encoding* (23, 24) to encode the conditional attributes, i.e., age and heath state (diagnosis of AD). The effectiveness of Fourier encoding has been experimentally shown in Tancik et al. (23), Mildenhall et al. (24). We also compared the generative model using Fourier v.s. Ordinal encoding using the quantitative metrics briefly introduced in Section 2.1, as presented in Table 1. We observe that the generator using *Fourier encoding* achieves very similar quantitative results as the generator using *ordinal encoding*, demonstrating effectiveness of Fourier encoding to encode age and health status.

**Table 1:** Quantitative results of brain ageing model using *ordinal encoding* and *Fourier encoding*.

Encoding	SSIM	PSNR	MSE	PAD
Ordinal encoding	$0.79_{0.06}$	$26.1_{2.6}$	$0.042_{0.006}$	$4.2_{3.9}$
Fourier encoding	$0.79_{0.08}$	$25.9_{2.7}$	$0.043_{0.009}$	$4.1_{3.7}$

For detail of the evaluation metrics please refer to Xia et al. (21), Section 4.

The key idea of Fourier encoding is to map low-dimensional vectors to a higher dimensional domain using a set of sinusoids. For instance, if we have a *d*-dimensional vector which is normalised into [0,1), $v\in [0,1{)}^{d}$, then the encoded vector can be represented as Tancik et al. (23):

(1) $$\begin{array}{rl} \gamma (v) & =[p_{1}\cos(2\pi b_{1}^{T}v),p_{1}\sin(2\pi b_{1}^{T}v),\ldots ,\\ & p_{m}\cos(2\pi b_{m}^{T}v),p_{m}\sin(2\pi b_{m}^{T}v)], \end{array}$$

where $b_{\;j}$ can be viewed as the Fourier basis frequencies, and $p_{\;j}^{2}$ the Fourier series coefficients.

In this work, the vector v represents the target age a and the health status (AD diagnosis), and d=2. In our experiments, we set $p_{\;j}^{2}=1$ for j=1,…,m, and $b_{\;j}$ are independently and randomly sampled from a Gaussian distribution, $b_{\;j}∼N(\mu_{scale}\;*\;I,0)$, where $\mu_{scale}$ is set to 10. We set m=100 and the resulting γ(v) is 200-dimensional. After encoding, the generator G takes the encoded vector γ(v) as input.

The use of Fourier encoding offers two advantages. First, Xia et al. (21) encoded age and health state into two vectors and had to use two MLPs to embed the encoded vectors into the model. This may not be a big issue when the number of factors is small. However, extending the generative model to be conditioned on tens or hundreds of factors will increase the memory and computation costs significantly. With Fourier encoding, we can encode all possible factors into a single vector, which offers more flexibility to scale the model to multiple conditional factors. Second, Fourier encoding allows us to compute the gradients with respect to the input vector v or certain elements of v, since the encoding process is differentiable. As such, we replace the *ordinal encoding* with *Fourier encoding* for all experiments. The generative model G takes v as input: $\hat{x}=G(x,v)$, where v represents target age and health state. Since we only change the target age a in this paper, we write the generative process as $\hat{x}=G(x,a)$ for simplicity.

### 2.3. Adversarial counterfactual augmentation

Suppose we have a conditional generative model G and a classification model C. The goal is to utilise G to improve the performance of C. To this end, we propose an approach consisting of three steps: *pre-training*, *hard sample selection* and *adversarial classification training*. A schematic of the adversarial classification training is presented in Figure 1. Algorithm 1 summarises the steps of the method. Below we describe each step in detail.

**Algorithm 1:** Adversarial counterfactual augmentation with a pre-trained *G*.

**Input**: Training set *D*_*train*_; hyperparameter *k, N*; a pre-trained *G; C*.
**Pre-training**:
1. Train the classifiers *C* on *D*_*train*_ (Eq. 2).
**Hard sample selection:**
2. Select *N* samples from *D*_*train*_ that result in the highest classification errors for *C*, denoted as *D*_*hard*_.
**Adversarial classification training:**
3. Randomly initialize target ages *a*, and obtain initial synthetic data.
**For *k* do**
4. Update *a* in the direction to maximize classification error (Eq 4). 5. Obtain synthetic images with *D*_*hard*_ and the updated *a*, denoted as *D*_*syn*_. 6. Update *C* to optimize Eq. 5 on *D*_*train*_ ∪ *D*_*syn*_ for one epoch.

#### 2.3.1. Pre-training

The generative model is pre-trained using the same losses as in Xia et al. (21) except that we use Fourier encoding to encode age and AD diagnosis. Consequently, we obtain a pre-trained G that can generate counterfactuals conditioned on given target ages a: $\hat{x}=G(x,a)$.

The classification model C is a VGG-based network (22) trained to predict the AD diagnosis from brain images, optimised by minimising:

(2) $$L_{\;pre\text{-}train}=E_{x∼X_{train},y∼Y_{train}}L_{s}(C(x),y),$$

where $L_{s}(\cdot )$ is a supervised loss (binary cross-entropy loss in this paper), x is a brain image, and y is its ground-truth AD label. To note that if the pre-trained G and C are available in practice, we could avoid the pre-training step.

#### 2.3.2. Hard sample selection

Liu et al. (25), Feldman and Zhang (26) suggested that training data samples have different *influence* on the training of a supervised model, i.e., some training data are *harder* for the task and are more *effective* to train the model than others. Liu et al. (25) propose to up-sample, i.e. duplicate, the *hard* samples as a way to improve the model performance. Based on these observations, we propose a similar strategy to Liu et al. (25) to select these *hard* samples: we record the classification errors of all training samples for the pre-trained C and then select N=100 samples with the highest errors. The selected *hard* samples are denoted as $D_{hard}$: $\left\{X_{hard},Y_{hard}\right\}$.

#### 2.3.3. Adversarial classification training

Bowles et al. (14), Frid-Adar et al. (27), Dar et al. (28) augmented datasets by randomly generating a number of synthetic data with pre-trained generators. Similar to training samples, some synthetic data could be more *effective* for downstream tasks than others. Here we assume that if a synthetic data sample is *hard*, then it is more *effective* for training. We propose an adversarial game to find the *hard* synthetic data to boost C.

Specifically, let us first define the classification loss for synthetic data as:

(3) $$L_{C}=E_{x∼X_{hard},y∼Y_{hard}}L_{s}(C(\hat{x}),y),$$

where $\hat{x}$ is a generated sample conditioned on the target age a: $\hat{x}=G(x,a)$, and y is the ground-truth AD label for x. Here we assume that changing target age does not change the AD status, thus x and $\hat{x}$ have the same AD label.

Since the encoding of age a is differentiable (see Section 2.2), we can obtain the gradients of $L_{C}$ with respect to a as: $\nabla_{a}L_{C}=\nabla_{a}[L_{s}(C(G(x,a)),y)]$, and update a in the direction of *maximising* $L_{C}$ by: $\tilde{a}=a+\gamma_{a}\nabla_{a}L_{C}$, where $\gamma_{a}$ is the step size (learning rate) for updating a. Formally, the optimization function of a can be written as:

(4) $$L_{1}=\max_{a}E_{x∼X_{hard},y∼Y_{hard}}L_{s}(C(\hat{x}),y).$$

Then we could obtain a set of synthetic data using the updated $\tilde{a}$: ${\hat{x}}_{syn}=G(x,\tilde{a})$ where $x∼X_{hard}$, denoted as $D_{syn}\;:\;\{X_{syn},Y_{syn}\}$.

The classifier C is updated by optimising:

(5) $$L_{2}=\min_{C}E_{x∼X_{combined},y∼Y_{combined}}L_{s}(C(x),y),$$

where $D_{combined}$: $\left\{X_{combined},Y_{combined}\right\}$ is a combined dataset consisting of the training dataset and synthetic dataset: $\left\{X_{combined},Y_{combined}\}=\{X_{train}\cup X_{syn},Y_{train}\cup Y_{syn}\right\}$. Similar to Liu et al. (25), we update C on $D_{combined}$ instead of $D_{syn}$ as we found updating C only on $D_{syn}$ can cause catastrophic forgetting (29).

The adversarial game is formulated by alternatively and iteratively updating a and classifier C via Eqs. 4 and 5, respectively. In practice, to prevent a from going to unsuitable ages, we clip it to be in [60, 90] after every update.

#### 2.3.4. Updating a vs. updating G

Note here the adversarial game is formulated between a and C, instead of G and C. This is because training G against C allows G to change its latent space without considering image quality, and the output of G could be unrealistic. Please refer to Section 4.1.2 for more details and results.

#### 2.3.5. Counterfactual augmentation vs. conventional augmentation

Here we choose to augment data counterfactually instead of applying conventional augmentation techniques. This is because that the training and testing data are already pre-processed and registered to MNI 152, and in this case conventional augmentations do not introduce helpful variations. Please refer to Section 4.1.3 for more details and results.

### 2.4. Adversarial classification training in a *continual learning* context

Most previous works (14, 27, 28, 30–32) that used pre-trained deep generative models for augmentation focused on generating a large number of synthetic samples, and then merged the synthetic data with the original dataset and trained the downstream task model (e.g. a classifier) on this augmented dataset. However, this requires training the task model from scratch, which could be inconvenient. Imagine that we are given a pre-trained classifier, and we have a generator at hand which may or may not be pre-trained on the same dataset. We would like to use the generator to improve the classifier, or transfer the *knowledge* learnt by the generator to the classifier. The strategy of previous works is to use the generative model to produce a large amount of synthetic data that cover the *knowledge* learnt by the generator, and then train the classifier on both real and synthetic data from scratch, which would be expensive. However, in this work, we consider the task of transferring knowledge from the generator to the classifier in the continual learning context, by considering synthetic data as new samples. We want the classifier to learn new *knowledge* from these synthetic data without forgetting what it has learnt from the original classification training set. We will show how our approach can help in the continual learning context.

In Section 2.3, after we obtain the synthetic set $D_{syn}$, we choose to update the classifier C on the augmented dataset $D_{syn}\cup D_{train}$, instead of $D_{syn}$ (stage 6 in Algorithm 1). This is because re-training the classifier only on the $D_{syn}$ would result in *catastrophic forgetting* (29), i.e. a phenomenon where deep neural networks tends to forget what it has learnt from previous data when being trained on new data samples. To alleviate catastrophic forgetting, efforts have been devoted to developing approaches to allow artificial neural networks to learn in a sequential manner (33, 34). These approaches are known as *continual learning* (33, 35, 36), *lifelong learning* (37, 38), *sequential learning* (39, 40), or *incremental learning* (41, 42). Despite different names and focuses, the main purpose of these approaches is to overcome catastrophic forgetting and to learn in a sequential manner.

If we consider the generated data as new samples, then the update of the pre-trained classifier C can be viewed as a *continual learning* problem, i.e. how to learn *new* knowledge from the synthetic set $D_{syn}$ without forgetting *old* knowledge that is learnt from the original training data $D_{train}$. To alleviate catastrophic forgetting, we re-train the classifier on both the synthetic dataset $D_{syn}$ and the original training dataset $D_{train}$. This strategy is known as *memory replay* in continual learning (43, 44) and was also used in other augmentation works (25). The key idea is to store previous data in a *memory buffer* and *replay* the saved data to the model when training on new data. However, it could be expensive to store and revisit all the training data, especially when the data size is large (44). In Section 4.2, we perform experiments where we only provide a portion (M%) of training data to the classifier when re-training with synthetic data (to simulate the *memory buffer*). In this case, we only create synthetic data from the *memory bank.* We want to see whether *catastrophic forgetting* would happen or not when only a portion (M%) of training data is provided, and if so, how much it affects the test accuracies. Algorithm 2 summarises the steps of the method in the *continual learning* context.

**Algorithm 2:** Adversarial classification learning with D_*store*_.

**Input**: Training dataset *D*_*train*_; hyperparameter *M, N, k;* a pre-trained generator *G*; a pre-trained classifier model *C*.
**Construct** *D*_*store*_:
1. Randomly select *M%* data from *D*_*train*_, denoted as *D*_*store*_.
**Hard sample selection:**
2. Select *N* samples from *D*_*store*_ that result in the highest classification errors for *C*, denoted as *D*_*hard*_.
**Adversarial training:**
3. Randomly initialize target ages *a*, and obtain initial synthetic data.
**For** *k* **do**
4. Update *a* in the direction to maximize classification error (Equation 4). 5. Obtain synthetic images with *D*_*hard*_ and the updated *a*, denoted as *D*_*syn*_. 6. Update *C* to minimize the classification error on *D*_*store*_ ∪ *D*_*syn*_ (Equation 5).

## 3. Experimental setup

### 3.1. Data

We use the ADNI dataset (45) for experiments. We select 380 AD and 380 CN (control normal) T1 volumes between 60 and 90 years old. We split the AD and CN data into training/validation/testing sets with 260/40/260 volumes, respectively. All volumetric data are skull-stripped using DeepBrain^5^, and linearly registered to MNI 152 space using FSL-FLIRT (46). We normalise brain volumes by clipping the intensities to $\left[0,V_{99.5}\right]$, where $V_{99.5}$ is the 99.5% largest intensity value within each volume, and then rescale the resulting intensities to the range [−1, +1]. We select the middle 60 axial slices from each volume and crop each slice to the size of [208,160], resulting in 31,200 training, 4,800 validation and 9,600 testing slices.

### 3.2. Implementation

The generator is trained the same way as in Xia et al. (21), except we replace *ordinal encoding* with *Fourier encoding*. We pre-train the classifier for 100 epochs. The experiments are performed using Keras and Tensorflow. We train pre-trained classifiers C with Adam with a learning rate of 0.00001 and decay of 0.0001. During adversarial learning, the step size of a is tuned to be 0.01, and the learning rate for C is 0.00001. The experiments are performed using a NVIDIA Titan X GPU.

### 3.3. Comparison methods

We compare with the following baselines:

1. *Naïve*: We directly use the pre-trained C for comparison as the *lower bound*.
2. *RSRS*: Random Selection + Random Synthesis. We randomly select N=100 samples from the training set $D_{train}$, denoted as $D_{rand}$, and then use the generator G to randomly generate $N_{synthesis}=5$ synthetic samples for each sample in $D_{rand}$, denoted as $D_{syn}$. Then we train the classifier on the combined dataset $D_{train}\cup D_{syn}$ for k=5 steps. This is the typical strategy used by most previous works (14, 27, 28).
3. *HSRS*: Hard Selection + Random Synthesis. We select N=100 hard samples from $D_{train}$ based on their classification errors of C, denoted as $D_{hard}$, and then use the generator G to randomly generate $N_{synthesis}=5$ synthetic samples for each sample in $D_{hard}$, denoted as $D_{syn}$. Then we train the classifier on the combined dataset $D_{train}\cup D_{syn}$ for k=5 steps.
4. *RSAT*: Random Selection + Adversarial Training. We randomly select N=100 samples from the training set $D_{train}$, denoted as $D_{rand}$, and then use the adversarial training strategy to update the classifier C, as described in Section 2.3. The difference between RSAT and our approach is that we select hard samples for generating counterfactuals, while RSAT uses random samples.
5. *JTT*: Just Train Twice (25) record samples that are misclassified by the pre-trained classifier, obtaining an error set. Then they construct an oversampled dataset $D_{up}$ that contain examples in the error set $\lambda_{up}$ times and all other examples once. Finally, they train the classifier on the oversampled dataset $D_{up}$. In this paper, we set $\lambda_{up}=2$ as we found large $\lambda_{up}$ results in bad performance. We also found the original learning rate (0.01) used for the second training stage results in very bad performance and set it to be 0.00001.

## 4. Results and discussion

### 4.1. Improving the performance of classifiers

#### 4.1.1. Comparison with baselines

We first compare our method with baseline approaches by evaluating the test accuracy of the classifiers. We set N=100 and k=5 in experiments. We pre-train C for 100 epochs and G as described in Section 3. The weights of the pre-trained C and the pre-trained G are the same for all methods. For a fair comparison, the total number of synthetically generated samples is fixed to 500 for *RSRS*, *HSRS*, *RSAT* and our approach. For *JTT*, there are 2,184 samples mis-classified by C and oversampled. We initialize a randomly between real ages of original brain images x and maximal age (90 yrs old).

From Table 2 we can observe that our proposed procedure achieves the best overall test accuracy, followed by baseline *RSAT*. This demonstrates the advantage of adversarial training between the conditional factor (target age) a and the classifier. On top of that, it shows that selecting *hard* examples for creating augmented synthetic results helps, which is also demonstrated by the improvement of performance of *HSRS* over *Naïve*. We also observe that *JTT* (25) improves the classifier performance over *Naïve*, showing the benefit of up-sampling *hard* samples. In contrast, baseline *RSRS* achieves the lowest overall test accuracy, even lower than that of *Naïve*. This shows that randomly synthesising counterfactuals from randomly selected samples could result in synthetic images that are harmful to the classifier.

**Table 2:** Average test accuracies of models trained via our procedure and baselines.

Acc %	CN	AD	All
Age group	60–70 yrs	70–80 yrs	80–90 yrs	60–70 yrs	70–80 yrs	80–90 yrs	Overall
Test group size	1,540	1,600	1,660	1,720	1,540	1,540	9,600
Naïve	85.2	91.5	*70.7*	92.5	94.2	**97.1**	88.4
RSRS	86.0	90.4	*73.8*	87.3	95.1	90.0	87.0
HSRS	85.6	91.1	*80.4*	89.8	93.8	96.9	89.5
RSAT	86.1	93.1	*81.5*	91.8	96.0	95.7	90.6
JTT	83.9	**94.2**	*80.1*	**92.8**	90.8	93.7	89.2
Proposed	**86.4**	93.7	***83.4***	91.5	**96.5**	95.7	**91.1**

We first present the average test accuracies for different age groups with AD (column 2–4) or CN (column 5–7) and then present the average test accuracies for the whole testing set (column 8). For each method, the *worst-group* performance is shown in *italic*. For each age group, i.e. each column, the **best** performance is shown in **bold**. We also report the number of testing images for each age group.

**Figure 2:**
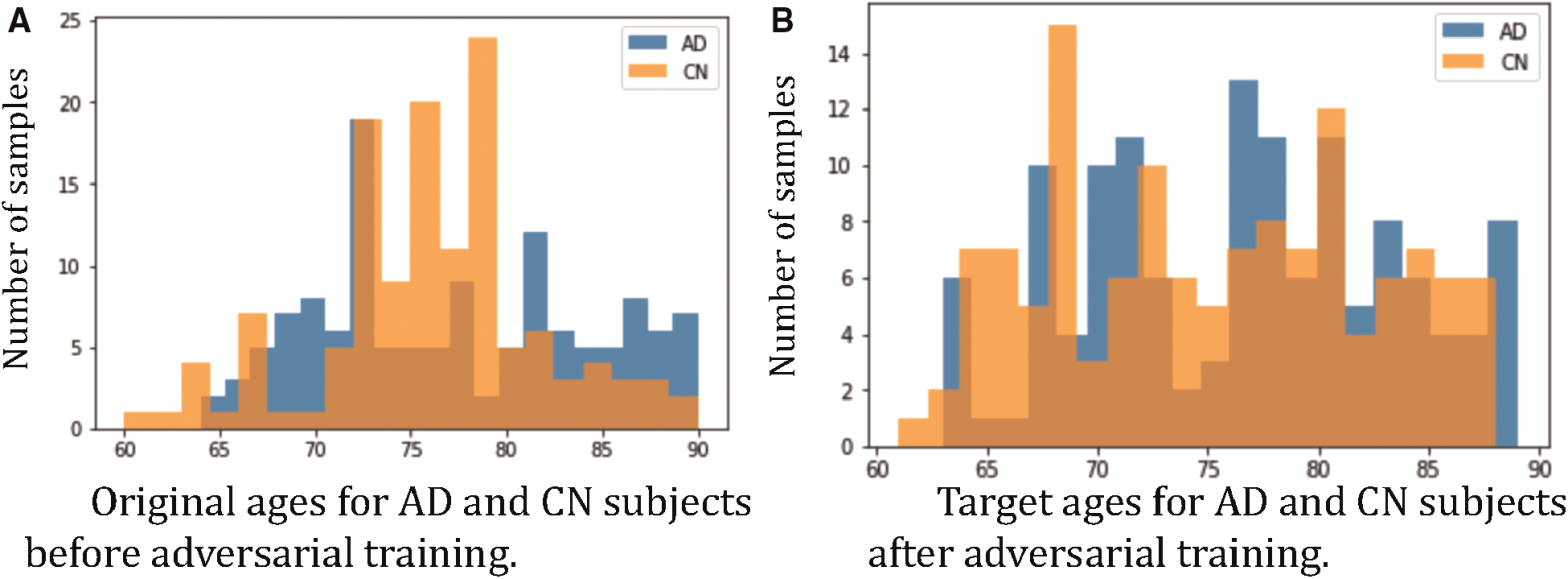
Histograms of ages of subjects before and after adversarial learning. We can observe that adversarial training aims to balance the data.

Furthermore, we observe that for all methods, the *worst-group* performances are achieved on the 80–90 CN group. A potential reason could be: as age increases, the brains shrink, and it is harder to tell if the ageing pattern is due to AD or caused by normal ageing. Nevertheless, we observe that for this *worst group*, our proposed method still achieves the best performance, followed by RSAT. This shows that adversarial training can be helpful to improve the performance of the classifier, especially for *hard* groups. The next best results are achieved by *HSRS* and *JTT*, which shows that finding hard samples and up-sampling or augmenting them was helpful to improve the *worst-group* performance. We also observe the improvement of *worst-group* performance for *RSRS* over *Naïve*, but the improvement is small compared to other baselines. Figure 2 presents histograms of original ages for training subjects and the target ages after adversarial training, where we can see how the adversarial training aims to balance the data.

We also report the *precision* and *recall* for all methods, as presented in Table 3. We can observe that our approach achieves the highest overall precision and recall results.

**Table 3:** The test precision and recall values for all methods.

Age range	60–70	70–80	80–90	Overall	60–70	70–80	80–90	Overall
Metrics	Precision	Recall
Naive	0.875	0.914	0.761	0.842	0.925	0.942	**0.971**	**0.945**
RSRS	0.874	0.905	0.768	0.844	0.873	0.951	0.900	0.906
HSRS	0.874	0.910	0.826	0.866	0.898	0.938	0.969	0.933
RSAT	0.881	0.930	0.832	0.877	0.918	0.960	0.957	0.943
JTT	0.865	**0.938**	0.822	0.868	**0.928**	0.908	0.960	0.924
Proposed	**0.883**	0.936	**0.848**	**0.885**	0.915	**0.965**	0.965	**0.945**

We first present the precision for different age groups (column 2-4) and all testing data (column 5), and then present the recall for different age groups (column 6–8) and all testing data (column 9). For each group, the **best** results are shown in **bold**.

In summary, the quantitative results show that it is helpful to find and utilise *hard* counterfactuals for improving the classifier.

#### 4.1.2. Train *G* against *C*

We choose to formulate an adversarial game between the conditional generative factor a (the target age) and the classifier C, instead of between the generator G and the classifier C. This is because we are concerned that an adversarial game between G and C could result in unrealistic outputs of G. In this section, we perform an experiment to investigate this.

Specifically, we define an optimization function:

(6) $$L_{G}=\max_{G}E_{x∼X_{train},y∼Y_{train}}L_{s}(C(G(x,a)),y),$$

where we aim to train G in the direction of maximising the loss of the classifier C on the synthetic data G(x,a).

After every update of G, we construct a synthetic set $D_{syn}$ by generating 100 synthetic images from $D_{train}$, and update C on $D_{train}\cup D_{syn}$ via Equation 5. The adversarial game G vs. C is formulated by alternatively optimising Equations 6 and 5 for 10 epochs.

**Figure 3:**
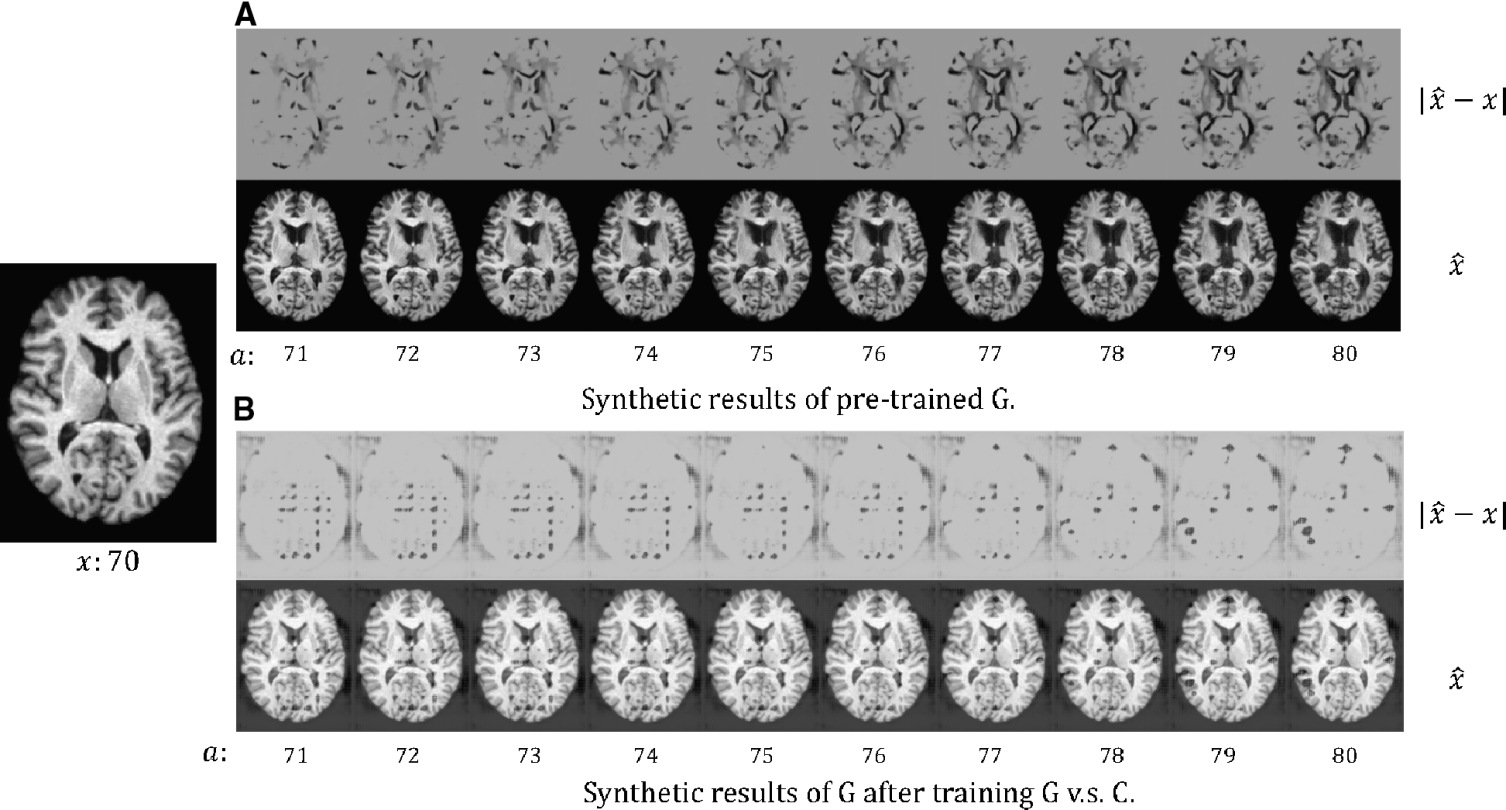
The synthetic results for a healthy (CN) subject x at age 70: (**A**) the results of the pre-trained G, i.e. before we train G against C; (**B**) the results of G after we train G against C. We synthesise aged images $\hat{x}$ at different target ages a. We also visualise the difference between x and $\hat{x}$, $\left|\hat{x}-x\right|$. For more details see text.

In Figure 3, we present the synthetic brain ageing progression of a CN subject before and after the adversarial training of G vs. C. We can observe that after the adversarial training, the generator G produces unrealistic results. This could be because there is no loss or constraint to prevent the generator G from producing low-quality results. The adversarial game only requires the generator G to produce images that are hard for the classifier C, and naturally, images of low quality would be hard for C. A potential solution could be to involve a GAN loss with a discriminator to improve the output quality, but this would make the training much more complex and require more memory and computations. We also measure the test accuracy of the classifier C after training G against C to be 81.6%, which is much lower than the *Naïve* method (88.4%) and our approach (91.1%) in Table 2. The potential reason is that C is misled by the unrealistic samples generated by G.

**Figure 4:**
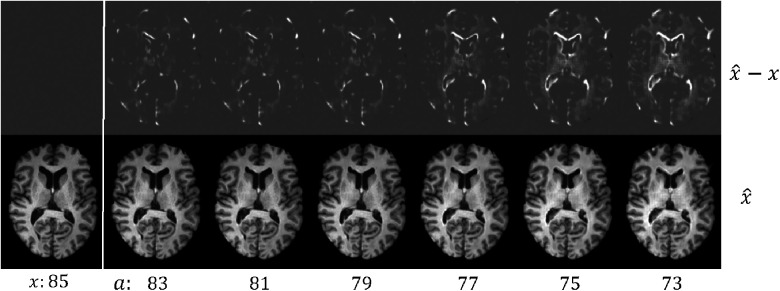
Example results of brain *rejuvenation* for an image (x) of a 85 year old CN subject. We synthesise *rejuvenated* images $\hat{x}$ at different target ages a. We also show the differences between $\hat{x}$ and x, $\hat{x}-x$. For more details see text.

#### 4.1.3. Effect of conventional augmentations for registered brain MRI data

In this section, we test the effect of applying several commonly used conventional augmentations, e.g. rotation, shift, scale and flip, to the training of the AD classifier. These are typical conventional augmentation techniques applied to computer vision classification task. Specifically, we train the classifier the same way as *Naïve*, except we augment training data with conventional augmentations.

Interestingly, we find that after applying rotation (range 10 degrees), shift (range 0.2), scale (range 0.2), and flip to augment the training data, the accuracy of the trained classifier drops from 88.4% to 71.6%. We then measure accuracies when trained with each augmentation to be 74.1% (rotation), 87.1% (shift), 82.9% (scale), and 87.8% (flip). We also trained the classifier with random gamma correction (gamma ranges from 0.2 to 1.8), and the resulting test accuracy is 84.4%. This could be because both training and testing data are already pre-processed, including registered to MNI 152 and contrast normalisation, and these conventional augmentations do not introduce helpful variations to the training data but distract the classifier from focusing on subtle differences between AD and CN brains.

We also tried to train the classifier with MaxUp (10) with conventional augmentations. The idea of MaxUp is to generate a small batch of augmented samples for each training sample and train the classifier on the *worst-performance* augmented sample. The overall test accuracy is 57.7%. This could be because that MaxUp tends to select the augmentations that distract the classifier from focusing on subtle AD features the most.

The results with conventional augmentations (+MaxUp) suggest that for the task of AD classification, when training and testing data are pre-processed well, conventional data augmentation techniques seem to not help improve the classification performance. Instead, these augmentations distract the classifier from identifying subtle changes between CN and AD brains. By contrast, the proposed procedure augment data in terms of semantic information, which could alleviate data imbalance and improve classification performance.

### 4.2. Adversarial counterfactual augmentation in a *continual learning* context

#### 4.2.1. Results when re-training with a portion (M%) of training data

Suppose we have a pre-trained classifier C and a pre-trained generator G, and we want to improve C by using G for data augmentation. However, after pre-training, we only store M% (M∈(0,100]) of the training dataset, denoted as $D_{store}$. During the adversarial training, we synthesise N samples using the generator G, denoted as $D_{syn}$. Then we update the classifier C on $D_{store}\cup D_{syn}$, using Equation 5 where $D_{combined}=D_{store}\cup D_{syn}$. The target ages are initialised and updated the same way as in Section 4.1. Algorithm 2 illustrates the procedure in this section.

Table 4 presents the test accuracies of our approach and baselines when M changes. For *Naïve-100*, the results are then same as in Table 2. For JTT, the original paper Liu et al. (25) retrained the classifier using the whole training set. Here we first randomly select M% training samples as $D_{store}$ and find misclassified data $D_{mis}$ within $D_{store}$ to up-sample, then we retrain the classifier on the augmented set. We can observe that when M decreases, *catastrophic forgetting* happens for all approaches. However, our method suffers the least from catastrophic forgetting, especially when M is small. With M=20% of training data for retraining, our approach achieves better results than *Naïve*. This might be because the adversarial training between a and C tries to detect what is missing in $D_{store}$ and tries to recover the missing data by updating a towards those directions. We observe that *RSAT* achieves the second best results, only slightly worse than the proposed approach. Moreover, *HSRS* and *JTT* are more affected by catastrophic forgetting and achieve worse results. This might be because the importance of selecting *hard* samples declines as M decreases, since the $D_{store}$ becomes smaller.

**Table 4:** Test accuracies of our approach and baselines when the ratio of the size $D_{store}$ vs. the size of $D_{train}$ changes.

Acc %	M%
Methods	1	10	20	50	100
Naïve	N/A	N/A	N/A	N/A	88.4
HSRS	75.6	81.4	84.5	87.4	89.5
RSAT	84.2	85.8	87.2	88.6	90.6
JTT	77.3	82.3	85.1	88.1	89.2
Proposed	84.8	86.8	88.5	89.4	91.1

We can observe the decreases of test accuracies when M decreases, which was due to the effect of *catastrophic forgetting*.

These results demonstrate that our approach could alleviate *catastrophic forgetting*. This could be helpful in cases where we want to utilise generative models to improve pre-trained classifiers (or other task models) without *revisiting* all the training data (a *continual learning* context).

#### 4.2.2. Results when number of samples used for synthesis (N) changes

We also performed experiments where we changed N, i.e. the number of samples used for generating counterfactuals. Specifically, we set M=1, i.e. only 1% of original training data are used for re-training C, to see how many synthetic samples are needed to maintain good accuracy, especially when there are only a few training data stored in $D_{store}$. This is to see how *efficient* the synthetic samples are in terms of training C and alleviating *catastrophic forgetting*. The results are presented in Table 5.

**Table 5:** Test accuracies when N changes (M=1) of our approach and baselines.

Acc %	*N*
Methods	1 (0.64%)	10 (6.4%)	50 (32.1%)	100 (64.1%)
HSRS	65.4	71.0	73.4	75.6
RSAT	81.3	82.1	83.2	84.2
Proposed	82.1	82.9	84.1	84.6

We also show the percentage of N vs. the total number of $D_{store}$.

From Table 5, we can observe that the best results are achieved by our method, followed by *RSAT*. Even with only one sample for synthesis, our method could still achieve a test accuracy of 80%. This is probably because the adversarial training of a *vs.* C guides G to generate *hard* counterfactuals, which are efficient to train the classifier. The results demonstrate that our approach could help alleviate *catastrophic forgetting* even with a small number of synthetic samples used for augmentation. This experiment could also be viewed as a measurement of the *sample efficiency*, i.e. how efficient a synthetic sample is in terms of re-training a classifier.

### 4.3. Can the proposed procedure alleviate *spurious correlations*?

*Spurious correlation* occurs when two factors appear to be correlated to each other but in fact they are not (47). Spurious correlation could affect the performance of deep neural networks and has been actively studied in computer vision field (25, 48–51) and in medical imaging analysis field (52, 53). For instance, suppose we have an dataset of *bird* and *bat* photos. For *bird* photos, most backgrounds are *sky*. For *bat* photos, most backgrounds are *cave*. If a classifier learns this spurious correlation, e.g. it classifies a photo as *bird* as long as the background is *sky*, then it will perform poorly on images where *bats* are flying in the *sky*. In this section, we investigate if our approach could correct such *spurious correlations* by changing a to generate hard counterfactuals.

Here we create a dataset where 7860 images between 60 and 75 yrs old are AD, and 7,680 images between 75 and 90 yrs old are healthy, denoted as $D_{spurious}$. This is to construct a *spurious correlation*: young→AD and old→CN (in reality older people have higher chances of getting AD (54)). Then we pre-train C on $D_{spurious}$. The brain ageing model proposed in Xia et al. (21) only considered simulating *ageing* process, but did not consider brain *rejuvenation*, i.e., the reverse of ageing. To utilise old CN data, we pre-train another generator in the *rejuvenation* direction, i.e.,generating *younger* brain images from old ones. As a result, we obtain two generators that are pre-trained on $D_{train}$, denoted as $G_{ageing}$ and $G_{rejuve}$, where $G_{rejuve}$ is trained to simulate the *rejuvenation* process. Figure 4 shows visual results of $G_{rejuve}$. After that, we select 50 CN and 50 AD *hard* images from $D_{spurious}$, denoted as $D_{hard}$ and perform the adversarial classification training using $G_{rejuve}$ for *old CN* samples and $G_{ageing}$ for *young AD* samples. The target ages a are initialized as real ages of x.

After we obtain $G_{ageing}$ and $G_{rejuvenation}$, we select 50 CN and 50 AD images from $D_{spurious}$ that result in highest training errors, denoted as $D_{hard}$. Note that the selected CN images are between 75 and 90 yrs old, and the AD images are between 60 and 75 yrs old. Then we generate synthetic images from $D_{hard}$ using $G_{rejuvenation}$ for old CN samples and $G_{ageing}$ for young AD samples. The target ages a are initialized as their ground-truth ages. Finally, we perform the adversarial training between a and the classifier C. Here we want to see if the adversarial training can detect the *spurious correlations* purposely created by us, and more importantly, we want to see if the adversarial training between a and C can *break* the spurious correlations.

Table 6 presents the test accuracies of our approach and baselines. For *Naïve*, we directly use the classifier C pre-trained on $D_{spurious}$. For *HSRS*, we randomly generate synthetic samples from $D_{hard}$ for augmentation. For *JTT*, we simply select mis-classified samples from $D_{spurious}$ and up-sample these samples.

**Table 6:** Test accuracies for our procedure and baselines when C pre-trained on $D_{spurious}$.

Acc %	CN	AD	
Methods	60–75 yrs	75–90 yrs	60–75 yrs	75–90 yrs	Overall
Naïve	*40.9*	81.6	**95.1**	45.7	67.0
HSRS	*60.7*	85.3	81.1	67.2	75.0
JTT	50.5	**88.4**	85.5	*40.7*	67.9
Proposed	***73.1***	83.4	81.5	**75.8**	**79.0**

We first present the average test accuracies for different age groups with CN diagnosis (column 2–3) or AD (column 4–5), and then present the average test accuracies for the whole testing set (column 6). For each method, the *worst-group* performance is shown in *italic*. For each age group, i.e. each column, the **best** performance was shown in **bold**. For more details see text.

We can observe from Table 6 that the pre-trained C on $D_{spurious}$ (*Naïve*) achieves much worse performance (67.0% accuracy) compared to that of Table 2 (88.4% accuracy). Specifically, it tends to misclassify *young CN* images as AD and misclassify *old AD* images as CN. This is likely due to the spurious correlations that we purposely create in $D_{spurious}$: young→AD and old→CN. We notice that for *Naïve*, the test accuracies of AD groups are higher than that of CN groups. This is likely due to the fact we have more AD training data, and the classifier is biased to classify a subject to AD. This can be viewed as another *spurious correlation*. Overall, we observe that our method achieves the best results, followed by *HSRS*. This shows that the synthetic results generated by the generators are helpful to alleviate the effect of *spurious correlations* and improve downstream tasks. The improvement of our approach over *HSRS* is due to the adversarial training between a and C, which guides the generator to produce hard counterfactuals. We observe *JTT* does not improve the test accuracies significantly. A potential reason is that *JTT* tries to find “hard” samples in the training dataset. However, in this experiment, the “hard” samples should be *young CN* and *old AD* samples which do not exist in the training dataset $D_{spurious}$. By contrast, our procedure could guide G to generate these samples, and *HSRS* could create these samples by random chance.

**Figure 5:**
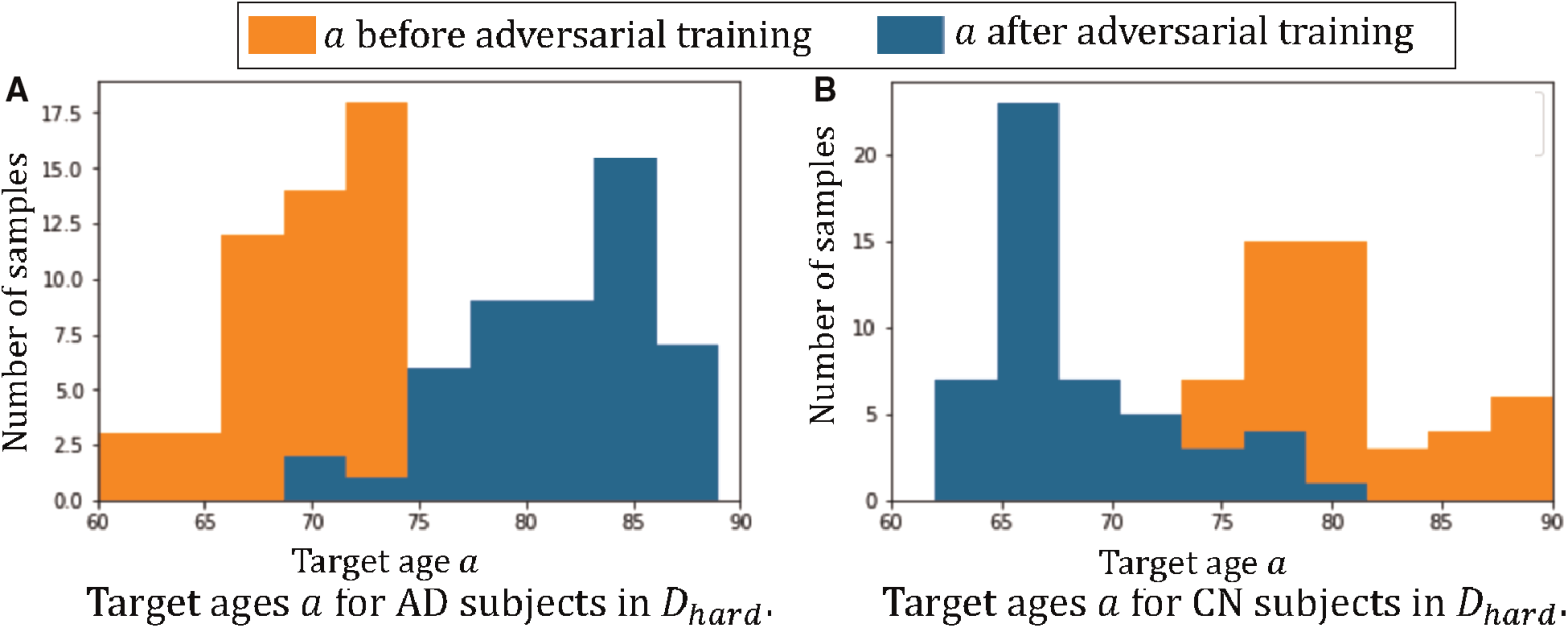
Histograms of target ages a before and after adversarial training: (**A**) the histogram of a for the 50 AD subjects in $D_{hard}$; (**B**) the histogram of a for the 50 CN subjects in $D_{hard}$. Here we show histograms of a before (in orange) and after (in blue) the adversarial training.

Figure 5 plots the histograms of the target ages a before and after the adversarial training. From Figure 5 we can observe that the adversarial training pushes a towards the *hard* direction, which could alleviate the spurious correlations. For instance, in $D_{spurious}$ and $D_{hard}$ the AD subjects are all in the *young* group, i.e. 60–75 yrs old, and the classifier learns the *spurious correlation*: young→AD, but in Figure 5A we can observe that the adversarial training learns to generate AD synthetic images in the range of 75–90 yrs old. These *old AD* synthetic images can help alleviate the spurious correlation and improve the performance of C. Similarly, we can observe a are pushed towards *young* for CN subjects in Figure 5B.

## 5. Conclusion

We presented a novel adversarial counterfactual scheme to utilise conditional generative models for downstream tasks, e.g. classification. The proposed procedure formulates an adversarial game between the *conditional factor* of a pre-trained generative model and the downstream *classifier*. The synthesis model used in this work uses two generators for ageing and rejuvenation. Others have shown that one model can handle both tasks albeit in another dataset and with less conditioning factors (55). We do highlight though that our approach is agnostic to the generator used and since could benefit from advances in (conditional) generative modelling. In this paper, we demonstrate that several conventional augmentation techniques are not helpful for registered MRI. However, there might be other heuristic-based augmentation techniques that will improve performance, and it is worth trying to combine our semantic augmentation strategy with such conventional augmentation techniques to further boost performance. The proposed adversarial counterfactual scheme could be applied to generative models that produced other types of counterfactuals rather than the ageing brain, e.g. the ageing heart (55, 56), future disease outcomes (57), existence of pathology (58, 59), etc. The way we updated the conditional factor (target age) could be improved. Instead of a continuous scalar (target age), we can consider extending the proposed adversarial counterfactual augmentation to update other types of conditional factors, e.g., discrete factor or image. The strategy that we used to select *hard* samples may not be the most effective and could be improved.

## Data Availability

Publicly available datasets were analyzed in this study. This data can be found here: https://adni.loni.usc.edu.

## Footnotes

^1^An example could be when training and testing brain MRI data are already well-registered, traditional augmentations, e.g. rotation, shift, etc., on the training data will hurt the performance of the trained model on testing data. See Section 4.1.3 for more details.

^2^Code is available at https://github.com/xiat0616/BrainAgeing

^3^Deep models continuously learn based on input of new data while preserving previously learnt knowledge.

^4^A popular deep learning neural network that has widely been used for classification.

^5^ https://github.com/iitzco/deepbrain
